# Proteomic and metabolomic analyses uncover integrative mechanisms in *Sesuvium portulacastrum* tolerance to salt stress

**DOI:** 10.3389/fpls.2023.1277762

**Published:** 2023-11-28

**Authors:** Dingding Cao, Wenbin Zhang, Nan Yang, Ziling Li, Chaoyue Zhang, Dan Wang, Guiping Ye, Jianjun Chen, Xiangying Wei

**Affiliations:** ^1^ Fujian Key Laboratory on Conservation and Sustainable Utilization of Marine Biodiversity, Fuzhou Institute of Oceanography, College of Geography and Oceanography, Minjiang University, Fuzhou, China; ^2^ Mid-Florida Research and Education Center, Department of Environmental Horticulture, Institute of Food and Agricultural Sciences, University of Florida, Apopka, FL, United States

**Keywords:** salt tolerance, *Sesuvium portulacastrum*, proteomics, metabolomics, mechanism

## Abstract

**Introduction:**

Salt stress is a major constraint affecting crop productivity worldwide. Investigation of halophytes could provide valuable information for improving economically important crops to tolerate salt stress and for more effectively using halophytes to remediate saline environments. *Sesuvium portulacastrum L.* is a halophyte species widely distributed in tropical and subtropical coastal regions and can absorb a large amount of sodium (Na). This study was to analyze *S. portulacastrum* responses to salt stress at morphological, physiological, proteomic, and metabolomic levels and pursue a better understanding of mechanisms behind its salt tolerance.

**Methods:**

The initial experiment evaluated morphological responses of S. portulacastrum to different concentrations of NaCl in a hydroponic system, and subsequent experiments compared physiological, proteomic, and metabolomic changes in *S. portulacastrum* after being exposed to 0.4 M NaCl for 24 h as immediate salt stress (IS) to 14 days as adaptive salt stress (AS). Through these analyses, a working model to illustrate the integrative responses of *S. portulacastrum* to salt stress was proposed.

**Results:**

Plants grown in 0.4 M NaCl were morphologically comparable to those grown in the control treatment. Physiological changes varied in control, IS, and AS plants based on the measured parameters. Proteomic analysis identified a total of 47 and 248 differentially expressed proteins (DEPs) in leaves and roots, respectively. KEGG analysis showed that DEPs, especially those occurring in roots, were largely related to metabolic pathways. Root metabolomic analysis showed that 292 differentially expressed metabolites (DEMs) occurred in IS plants and 371 in AS plants. Among them, 20.63% of upregulated DEMs were related to phenolic acid metabolism.

**Discussion:**

Based on the integrative analysis of proteomics and metabolomics, signal transduction and phenolic acid metabolism appeared to be crucial for *S. portulacastrum* to tolerate salt stress. Specifically, Ca2+, ABA, and JA signalings coordinately regulated salt tolerance in *S. portulacastrum*. The stress initially activated phenylpropanoid biosynthesis pathway through Ca2+ signal transduction and increased the content of metabolites, such as coniferin. Meanwhile, the stress inhibited MAPK signaling pathway through ABA and JA signal transduction, which promoted Na sequestration into the vacuole to maintain ROS homeostasis and enhanced *S. portulacastrum* tolerance to salt stress.

## 1 Introduction

Salinity is one of the most serious threats to crop production (Zhu, 2002; Flowers, 2004; Gupta and Huang, 2014). There are 100 million hectares of barren saline-alkali land and more than 2.4 million hectares of coastal tidal flats in China (Office, 1998; Wang et al., 2011). The primary ions in saline soils are Na^+^ and Cl^−^, which can disrupt the ion balance in plant cells, inhibit the absorption of K^+^, and cause nutritional deficiencies (Wang et al., 2012; Assaha et al., 2017). Excessive intake of Na^+^ has also been shown to replace Ca^2+^ in biofilms and destroy the membrane structure and semipermeable functions (Yuan et al., 2014; Stephan et al., 2016). Salt stress in plants includes membrane lipid peroxidation, increased reactive oxygen content, and the accumulation of osmotic adjustment substances. The content of the intermediate product malondialdehyde (MDA) represents the degree of membrane lipid peroxidation (Zhu, 2002). Salt stress also triggers the production of reactive oxygen species (ROS), which cause oxidation of lipids, proteins, DNA, and other cellular components. To reduce ROS damage, plants simultaneously activate antioxidant defense enzymes, including superoxide dismutase (SOD), catalase (CAT), glutathione reductase (GR), and ascorbic acid (APX), to mitigate oxidative stress (Mittova et al., 2003). Additionally, plants produce osmotic adjustment substances, including proline, soluble sugars and proteins, betaine, and polyamines, to increase osmotic balance at the cellular level to counteract salt stress effects (Pommerrenig et al., 2007; Boriboonkaset et al., 2013; Slama et al., 2015).

Halophytes can sustain their growth without obvious reductions in plant height, leaf number, or growth rate when grown in salt-contaminated environments. Physiologically, halophytes can maintain their chloroplast structure and function under salt stress (Hameed et al., 2021). Halophytic guard cells are less sensitive to salt stress than glycophytic guard cells, and the expression of salt-dependent genes in guard cells differs between glycophytes and halophytes, which is manifested by the activation of the ABA signaling pathway (Karimi et al., 2021). Genes responsible for salt stress are expressed differently and show post-translational modifications between glycophytes and halophytes, including salt overly sensitive (SOS), antiporter of Na^+^ (K^+^)/H^+^ (NHX), high-affinity potassium transporters (HKT), vacuolar H^+^ adenosine triphosphatase (V-H^+^-ATPase), and vacuolar H^+^ pyrophosphatases (V-H^+^-PPase) (Himabindu et al., 2016). Besides, glycophytes rely more on AMF (arbuscular mycorrhizal fungi) symbiosis than halophytes under the condition of salt stress (Pan et al., 2020). Thus, investigating the salt-tolerance mechanisms of halophytes could provide fundamental information on plant responses to salinity and improve crop tolerance to salt stress. *Sesuvium portulacastrum* L. is a perennial creeping fleshy herbaceous halophyte belonging to the Aizoaceae family. *S. portulacastrum* can absorb Na^+^ and transport it to shoots without substantial reduction in their growth; thus, it has been considered an appropriate plant for phytoremediation, desalination, and sand fixation in coastal regions (Patil et al., 2012; Lokhande et al., 2013). Recent studies have shown that tolerance of *S. portulacastrum* to salt stress is largely attributed to the regulation of water potential, accumulation of suitable mineral ions, and physiological and biochemical (osmotic regulation) adaptations regulated by the differential expression of relevant genes (Slama et al., 2006; Lokhande et al., 2013; Wang D. et al., 2022).

Plants adapt to salt stress through stress sensing, signal transduction, and metabolic changes (Mantri et al., 2012). Proteomic and metabolomic analyses provide an overview of plant responses at protein and metabolite levels. Kumari et al. (2015) reported that various functional classes of salt stress-responsive proteins were involved in salt tolerance, including those involved in photosynthesis, carbohydrate and energy metabolism, and signal transduction. More specifically, under salt stress, proteins such as SOD, dehydroascorbate reductase (DHAR), APX, Trx, Prx, GPX, GST, and 14-3-3 work together to scavenge reactive oxygen species and enhance salt tolerance (Kumari et al., 2015; Peng et al., 2019). Sodium compartmentalization by V-ATPase and accumulation of osmotic adjustment substances can balance osmosis between the vacuoles and cytoplasm (Yi et al., 2014; Ding et al., 2022). Additionally, Na can partially substitute for K and limit Ca and Mg uptake, causing a cellular imbalance of nutrients (Kumari et al., 2015). Metabolic studies have shown that proline and proline derivatives, glycine betaine, myo-inositol, sorbitol, and polyamines participate in salt tolerance in halophytes. A previous metabolomic study showed that after two weeks of salt stress, various metabolites, including flavonoid-related metabolites, phenylpropanoids and lignans, and alkaloids and amine compounds, were detected in the leaves and roots of three halophytes, *Salicornia brachiata*, *Suaeda maritima*, and *S. portulacastrum*, but species differed in their metabolites (Benjamin et al., 2019).

Recent studies on *S. portulacastrum* salt tolerance have largely focused on leaves (Slama et al., 2006; Chen et al., 2022b; Wang D. et al., 2022; Wang et al., 2023), but limited efforts have been made on the roots. Roots are the organ that first contacts Na, and how roots respond to Na is important for understanding how halophytes tolerate salt stress (Li et al., 2021). Morphologically, salt stress reduces root mass by reducing the growth of the main and lateral roots in soil (Julkowska et al., 2014; Farooq et al., 2015). Cell proliferation and expansion of rice primary roots are affected by ABA accumulation after salt stress (Huang et al., 2021). Root-localized stress triggers changes in xylem hydraulics, ROS, mobile peptides, and Ca^2+^, leading to shoot responses to salt stress (Choi et al., 2014). ROS and Ca^2+^ are rapid stress-induced signals that function closely with ABA pathways to protect cells through stomatal closure (Pitzschke et al., 2009).

The objectives of this study were to (1) analyze the morphological and physiological responses of *S. portulacastrum* shoots and roots to different concentrations of NaCl; (2) investigate the functional proteins of *S. portulacastrum* in response to short- and long-term salt stress by proteomics; (3) combine proteomic and metabolomic analyses to determine how *S. portulacastrum* root systems cope with salt stress; and (4) elucidate the mechanisms underlying salt tolerance in *S. portulacastrum*.

## 2 Materials and methods

### 2.1 Plant materials and salt adaptation experiments

Stem cuttings, a piece of stem including three nodes, were made from uniform plants of *S. portulacastrum* and rooted in a one-fourth Hoagland nutrient solution (Hoagland and Synder, 1933) for two weeks. After recording leaf number and plant height, rooted stem cuttings were transplanted into containers filled with the nutrient solution supplemented with 0 M, 0.2 M, 0.4 M, 0.6 M, and 0.8 M NaCl. Plants were grown in a growth room with a constant temperature of 24°C, a light intensity of approximately 400 μmol/m^2^/s, and a photoperiod of 16 h for four weeks. Leaves and roots were harvested and used for analyzing growth and physiological data.

To determine the immediate and adaptative stress responses of *S. portulacastrum* plants to salt stress, well-rooted stem cuttings were grown in the mentioned Hoagland solution amended with or without 0.4 M NaCl for 12 h, 24 h, 2 days, and 1 to 4 weeks. Based on growth rates and water contents, plants grown for 24 h were selected as immediate stress (IS) and 14 days as adaptive stress (AS) to salinity. Roots and leaves were sampled, stored at −80°C, and used for physiological, proteomic, and metabolomic analyses.

An additional experiment was conducted to evaluate the effects of coniferin on Arabidopsis root growth under salt stress. Seeds of Columbia ecotype were germinated on MS medium for a week, seedlings were transplanted to a medium containing 150 mM NaCl supplemented with 0.001 mM and 0.01 mM of coniferin. Coniferin concentrations were chosen based on previous studies (van Uden et al., 1991; Chun et al., 2019; Yoshioka et al., 2021). Plant root growth efficiency was calculated based on a previous study (Ehsaninia, 2023).

### 2.2 Physiological parameter analyses

#### 2.2.1 Water content

To determine the water content of *S. portulacastrum*, tissue fresh weights (FW) were recorded; the tissue was dried in an oven at 105°C for 15 min, and then dried at 80°C to a constant weight. After recording the dry weight (DW), the water content of the plant tissues was calculated using the following the formula: water content (%) = (FW − DW)/DW × 100.

#### 2.2.2 Relative growth rate

The initial fresh weight (W0) of the plants was determined at the beginning of the experiment and the final fresh weight (W1) of the plants was recorded at the end of the experiment. The relative growth rate (RGR) of the plants at a specific time was calculated using the following formula: RGR (%) = (W1 − W0)/W0 × 100. The RGR for root number, root length, plant height, and leaf number were calculated as described above. For example, RGR of root number = (final root number − initial root number)/initial root number × 100.

#### 2.2.3 Root vitality

Root viability was determined using the triphenyl tetrazolium chloride (TTC) method (McLeod et al., 2018). The experimental principle is that dehydrogenase in plant roots can reduce TTC to red triphenyl hydrazone (TTF), which is water insoluble. The absorbance of the solution at 485 nm was determined, and the reduction in TTC was calculated. Dehydrogenase activity was represented by the reduction in TTC and was used as an index of plant root vitality.

#### 2.2.4 Chlorophyll contents

Fresh leaves collected from different treatments were extracted using the ethanol extraction method (Ritchie, 2008). The content of chlorophyll *a* (chl *a*) and chl *b* were analyzed and calculated as follows: Chl *a* (mg/g) = (12.7 × OD663 − 2.69 × OD645) × V/(1,000 × W); Chl *b* (mg/g) = (22.9 × OD645 − 4.68 × OD663) × V/(1,000×W), and the total chl (mg/g) = chl *a* + chl *b*, where V was the volume of extract (mL) and W was the fresh weight (g) of the extracted leaf tissue.

#### 2.2.5 H_2_O_2_ content

The H_2_O_2_ assay kit was purchased from Nanjing Jiancheng Bioengineering Institute (Nanjing, China). All experimental procedures were performed in accordance with the manufacturer’s instructions. H_2_O_2_ reacts with molybdic acid to form a complex. The absorbance of this complex was measured at 405 nm, and the content of H_2_O_2_ was calculated based on the calculation formula (Kumar et al., 2021):


$$\begin{array}{c} {\text{H}}_{2}{\text{O}}_{2}(\text{mmol})=\frac{\text{OD}(\text{detection})-\text{OD}(\text{blank})}{\text{OD}(\text{standard})-\text{OD}(\text{blank})}\\ \times \text{Standard concentration}÷\text{Sample protein concentration} \end{array}$$

#### 2.2.6 MDA content

Malondialdehyde (MDA) is a product of lipid peroxidation and has been widely used as an indicator of oxidative stress (Davey et al., 2005). MDA assay kit was purchased from Nanjing Jiancheng Bioengineering Institute. All experimental procedures were carried out according to the manufacturer’s instructions. To remove the interference of the color reaction between soluble sugar and TBA, the solution absorbance was measured at 450 nm and 532 nm, respectively and the absorbance difference was used for calculating MDA content. The calculation formula is as follows:


$$\begin{array}{c} MDA(nmol/mgprotein)=\frac{OD\;(detection)-OD\;(control)}{OD(standard)-OD(blank)}\\ \times \frac{10\;mm\text{ol}/L}{Sample\;protein\;concentration(mg\;protein/mL)} \end{array}$$

#### 2.2.7 Proline content

Proline accumulation in plants is an indicator of disturbed physiological conditions. Proline reacts with an acidic ninhydrin solution to generate a red color. Thus, a colorimetric assay was used to determine proline content at an absorbance of 520 nm (Abrahám et al., 2010; Senthilkumar et al., 2021).

#### 2.2.8 CAT activity

For experiments at the cellular level, the CAT assay kit was purchased from the Nanjing Jiancheng Bioengineering Institute. All experimental procedures were performed in accordance with the manufacturer’s instructions. CAT activity was analyzed as described by Sun et al. (2020). The decomposition of H_2_O_2_ by CAT was quickly stopped by adding ammonium molybdate, and the remaining H_2_O_2_ reacted with ammonium molybdate to produce a light-yellow complex. The activity of CAT can be calculated by measuring the absorbance of the complex at 405 nm using the formula below:


$$\begin{array}{c} \text{CAT}(\mu \text{mol}/\text{mgprotein})=(\text{OD}(\text{control})-\text{OD}(\text{determination}))\\ \times \frac{271}{60\times 0.05\times \text{Sample protein concentration}(\text{mgprotein}/\text{L})} \end{array}$$

The reaction time was set for 60 s, and the amount of H_2_O_2_ decomposed by 1 µmol per second per milligram of protein was one unit (U) of activity.

#### 2.2.9 SOD activity

Superoxide dismutase (SOD) activity was quantified using the method described by Sun et al. (1988). The superoxide radical reduces nitroblue tetrazolium (NBT) to blue monoformazon, and the activity of NBT was calculated by measuring the absorbance of blue substance at 560 nm, which was used as the index of SOD activity.

#### 2.2.10 Data analysis

All data were analyzed using the statistical program SPSS 22.0 (IBM Corporation, Somers, NY). When significance was reached, means were separated by the least significant difference (LSD) at *P<*0.05, *P<*0.01, or *P<*0.001.

### 2.3 Proteomic analysis

#### 2.3.1 Sample preparation

Root and leaf samples from the IS and AS experiments were homogenized in a lysis buffer consisting of 2.5% SDS/100 mM Tris–HCl (pH 8.0) (Ding et al., 2022), followed by ultrasonication. After centrifugation, the proteins in the supernatant were precipitated by adding pre-cooled acetone four times. The protein pellet was dissolved in 8 M urea/100 mM Tris–HCl. After centrifugation, the supernatant was used for reduction reaction (10 mM DTT, 37°C for 1 h), followed by an alkylation reaction (40 mM iodoacetamide, room temperature/dark place for 30 min). Protein concentrations were measured using Bradford assay. Urea was diluted below 2 M using 100 mM Tris–HCl (pH 8.0). Trypsin was added at a ratio of 1:50 (enzyme:protein, w/w) for overnight digestion at 37°C. The next day, TFA was used to reduce the pH to 6.0 to end the digestion. After centrifugation (12,000*g*, 15 min), the supernatant was subjected to peptide purification using a Sep-Pak C18 desalting column. The peptide eluate was vacuum-dried and stored at −20°C for later use.

#### 2.3.2 LC–MS/MS analysis

The LC–MS/MS data acquisition was carried out on an Orbitrap Exploris 480 mass spectrometer coupled with an Easy-nLC 1200 system. Peptides were loaded through an auto-sampler and separated in a C18 analytical column (75 μm × 25 cm, C18, 1.9 μm, 100 Å). Mobile phases A (0.1% formic acid) and B (80% ACN and 0.1% formic acid) were used to establish the separation gradient. A constant flow rate was set at 300 nL/min. For DDA mode analysis, each scan cycl consisted of one full-scan mass spectrum (R = 60 K, AGC = 300%, max IT = 20 ms, scan range = 350 m/z–1,500 m/z) followed by 20 MS/MS events (R = 15 K, AGC = 100%, max IT = auto, cycle time = 2 s). The HCD collision energy is set to 30. The isolation window for precursor selection was set to 1.6 Da. The first target ion exclusion was set for 35 s (Yi et al., 2014).

#### 2.3.3 Database search and analysis of differentially abundant proteins

Raw data were analyzed with MaxQuant (V1.6.6) using the Andromeda database search algorithm (Tyanova et al., 2016). Spectral files were searched against the UniProt proteome database. LFQ mode was used for quantification. The MS1 match tolerance was set as 20 ppm for the first search and 4.5 ppm for the main search; the MS2 tolerance was set as 20 ppm; Match between runs was used for identification transfer. The search results were filtered with 1% FDR at both protein and peptide levels. Proteins denoted as decoy hits, contaminants, or only identified sites were removed, and the remaining identifications were used for further quantification analysis. The Kyoto Encyclopedia of Genes and Genomes (KEGG) and Gene Ontology (GO) databases were used to categorize and group the candidate proteins. The GO and pathways with a corrected *P* <0.05 were considered significant (Ding et al., 2022).

#### 2.3.4 RNA extraction and qRT-PCR

Total RNA was extracted from *S. portulacastrum* leaf and root samples using an OminiPlant RNA Kit (CWBIO, Beijing, China). Primers were designed with the Primer Premier 6 software (http://www.premierbiosoft.com/primerdesign). qRT-PCR was performed in CFX Connect (BIO-RAD) using the SYBR Green Master Mix, and amplified with 1 μL of cDNA, 5 μL of 2 × SYBR Green Master Mix, and 0.6 μM of each primer. The amplification program consisted of one cycle of 95°C for 15 min, and 40 cycles of 95°C for 15 s, and 58°C for 30 s. The relative expression of the target genes was normalized by comparison with the reference NADPH and analyzed using the 2^−ΔΔCT^ method (Livak and Schmittgen, 2001).

### 2.4 Metabolomic analysis

#### 2.4.1 Sample preparation and extraction

Root samples were dried using a vacuum freeze-dryer (Scientz-100F). The freeze-dried sample was crushed using a mixer mill (MM 400, Retsch) with a zirconia bead for 1.5 min at 30 Hz. 100 mg of lyophilized powder was dissolved in a 1.2 mL 70% methanol solution, vortexed 30 s every 30 min for six times, and placed in a refrigerator at 4°C overnight. Following centrifugation at 12,000 rpm for 10 min, the extracts were filtrated (SCAA-104, 0.22 μm pore size; ANPEL, Shanghai, China, http://www.anpel.com.cn/) before UPLC-MS/MS analysis (Kusano et al., 2011).

#### 2.4.2 UPLC conditions

The extracts were analyzed using an UPLC-ESI-MS/MS system (UPLC, SHIMADZU Nexera X2, https://www.shimadzu.com.cn/; MS, Applied Biosystems 4500 Q TRAP, https://www.thermofisher.cn/cn/zh/home/brands/applied-biosystems.html). The UPLC column was Agilent SB-C18 (1.8 µm, 2.1 mm ∗ 100 mm); the mobile phase consisted of solvent A, pure water with 0.1% formic acid, and solvent B, acetonitrile with 0.1% formic acid. Sample measurements were performed with a gradient program that employed the starting conditions of 95% A and 5% B. Within 9 min, a linear gradient to 5% A and 95% B was programmed, and a composition of 5% A and 95% B was kept for 1 min. Subsequently, a composition of 95% A and 5.0% B was adjusted within 1.1 min and kept for 2.9 min. The flow velocity was set at 0.35 mL per minute; the column oven was set to 40°C; and the injection volume was 4 μL. The effluent was alternatively connected to an ESI-triple quadrupole linear ion trap (QTRAP)-MS (Duan et al., 2013).

#### 2.4.3 ESI-Q TRAP-MS/MS

LIT and triple quadrupole (QQQ) scans were acquired on a triple quadrupole-linear ion trap mass spectrometer (Q TRAP), AB4500 Q TRAP UPLC/MS/MS System, equipped with an ESI Turbo Ion-Spray interface, and operated in positive and negative ion mode controlled by Analyst 1.6.3 software (AB Sciex). The ESI source operation parameters were as follows: ion source, turbo spray; source temperature 550°C; ion spray voltage (IS) 5,500 V (positive ion mode)/−4,500 V (negative ion mode); ion source gas I (GSI), gas II (GSII), and curtain gas (CUR) were set at 50, 60, and 25.0 psi, respectively; the collision-activated dissociation (CAD) was high. Instrument tuning and mass calibration were performed using 10 and 100 μmol/L polypropylene glycol solutions in the QQQ and LIT modes, respectively. QQQ scans were acquired in MRM experiments with a collision gas (nitrogen) set to the medium. DP and CE for individual MRM transitions were performed with further DP and CE optimization. A specific set of MRM transitions was monitored for each period according to the metabolites eluted within this period (Kumari et al., 2015).

#### 2.4.4 Principal component analysis

Unsupervised PCA was performed using the statistical function prcomp in R (www.r-project.org). The data were unit variance scaled before unsupervised PCA (Lever et al., 2017).

#### 2.4.5 Hierarchical cluster analysis and Pearson correlation coefficients

Hierarchical cluster analysis (HCA) results of samples and metabolites were presented as heatmaps with dendrograms, whereas Pearson correlation coefficients (PCC) between samples were calculated using the cor function in R and presented as only heatmaps. Both HCA and PCC were performed using the R package pheatmap. For HCA, the normalized signal intensities of metabolites (unit variance scaling) were visualized as a color spectrum.

#### 2.4.6 Selected differential metabolites

Significantly regulated metabolites between the groups were determined by VIP ≥1 and absolute log2FC (fold change) ≥1. VIP (variable important in the projection) values were extracted from OPLS-DA result, which also contain score plots and permutation plots, was generated using R package MetaboAnalystR. The data were log transformed (log2) and mean centered before OPLS-DA. To avoid overfitting, a permutation test (200 permutations) was performed.

#### 2.4.7 KEGG annotation and enrichment analysis

Identified metabolites were annotated using the KEGG Compound database (http://www.kegg.jp/kegg/compound/) and annotated metabolites were mapped to the KEGG Pathway database (http://www.kegg.jp/kegg/pathway.html) (Tanabe and Kanehisa, 2012). Pathways with significantly regulated metabolites were then fed into metabolite sets enrichment analysis (MSEA), and their significance was determined using hypergeometric test’s p-values.

## 3 Results

### 3.1 Morphological and physiological changes of *S. portulacastrum* in response to salt stress

**Figure 1:**
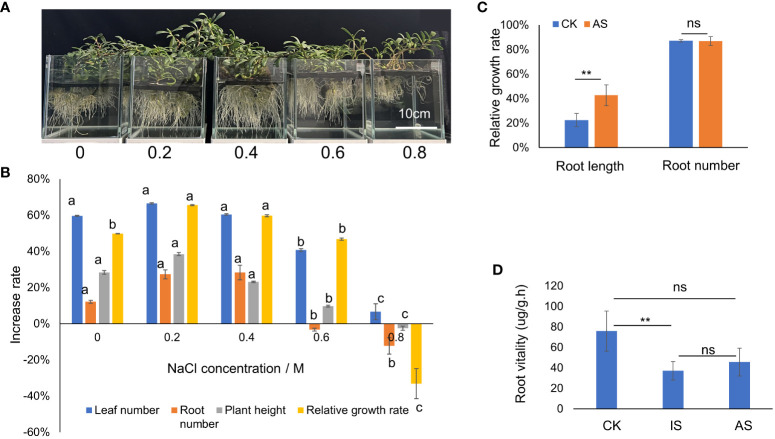
Growth responses of *S. portulacastrum* to different concentrations of NaCl in one-fourth Hoagland solution. **(A)** The depiction of plants grown in a hydroponic solution containing different concentrations of NaCl. **(B)** Changes in the growth rates of plants grown in different concentrations of NaCl. **(C)** The relative growth of root length and root numbers of control plants vs plants grown in 0.5 M NaCl for 14 days as adaptive stress (AS). **(D)** Root viability of control plants vs plants exposed to 0.5 M NaCl for 12 h as immediate stress and AS plants. Different letters above bars or ** indicate significant differences among treatments analyzed by LSD at *P<*0.01 level and ns (no significance) represents no significant difference.

The growth status of *S. portulacastrum* plants after exposure to different NaCl concentrations is shown in **Figure 1A**. The number of leaves and roots, plant heights, and relative growth rates after four weeks of growth are shown in **Figure 1B**. There were no significant differences in leaf and root numbers as well as plant heights when grown in the control, 0.2 and 0.4 M NaCl treatments, but these parameters substantially decreased when grown in 0.6 and 0.8 M NaCl. The relative growth rates of plants grown at 0.2 and 0.4 M NaCl were significantly higher (*P<*0.05) than plants grown in control and at 0.6 M but greatly reduced at 0.8 M (**Figure 1B**).

To determine plant responses to salt stress over time, plants were grown in the mentioned Hoagland solution supplemented with and without 0.4 M NaCl from 12 h to 3 weeks, respectively. The relative growth rate of plants exposed to 0.4 M NaCl for 12 h, 24 h, 2 days, and one week decreased but recovered from 2 to 3 weeks compared to the control treatment (**Supplementary Figure 1A**). Both leaf and stem water contents exhibited a downward trend, and root water content fluctuated, decreasing on day one, increasing on day two to maintain a plateau for up to a week, and then decreasing again (**Supplementary Figure 1B**). Based on the changes in relative growth rates and water contents, plant exposure to 0.4 M NaCl at 24 h was considered the immediate salt stress and for 2 weeks the adaptive salt stress. Compared with the control treatment, the root length of plants in the AS treatment increased significantly, but there was no significant difference in root number (**Figure 1C**). The root vitality of plants in both IS and AS was significantly decreased compared to that of the control (**Figure 1D**). There were no significant differences in chl *a* among plants in the control, IS, and AS treatments, but chl *b* was significantly higher in AS plants; thus, the total chlorophyll content of AS plants was significantly higher than that of the other treatments (**Table 1**).

**Table 1:** Concentrations of chlorophyll (chl) *a*, *b*, and their total in leaves of *S. portulacastrum* plants grown in one-fourth Hoagland solution with no Na (CK) and with 0.4 M NaCl for 24 h as immediate stress (IS) and 14 days as adaptive stress (AP), respectively.

Plants	Chl *a* (mg/g FW)	Chl *b* (mg/g FW)	Total chl (mg/g FW)
Control (CK)	0.6 ± 0.06 a	0.26 ± 0.02 b	0.86 ± 0.08 b
IS	0.64 ± 0.01 a	0.28 ± 0.02 b	0.92 ± 0.02 b
AS	0.63 ± 0.03 a	0.44 ± 0.05 a	1.07 ± 0.08 a

Data were mean ± standard error. Different letters after the means indicate significant difference among plants separated by LSD at P<0.05 level.

### 3.2 H_2_O_2_, MDA, and Pro contents and CAT and SOD activities in leaves and roots of *S. portulacastrum*

**Figure 2:**
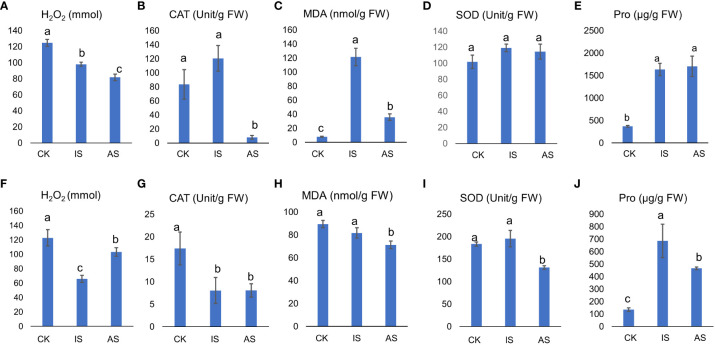
Physiological responses of *S. portulacastrum* plants grown in one-fourth Hoagland solution without NaCl as control (CK) and with 0.5 M NaCl for 12 h as immediate stress (IS) and 14 days as adaptive stress (AS). **(A, F)** H_2_O_2_ contents in leaves and roots; **(B, G)** CAT activities in leaves and roots; **(C, H)** MDA contents in leaves and roots; **(D, I)** SOD activities in leaves and roots; and **(E, J)** proline contents in leaves and roots, respectively. Unit, the amount of H_2_O_2_ decomposed by 1 µmol per second per milligram of protein is one unit of vitality. FW, fresh weight. Different letters above bars represent significant differences among treatments analyzed by LSD at *P* < 0.05 level.

The H_2_O_2_ content in *S. portulacastrum* leaves significantly decreased in IS and AS plants (**Figure 2A**), and CAT showed an increasing trend in IS plants but significantly decreased in AS plants (**Figure 2B**). The MDA content in the leaves of control plants was substantially lower than that of IS and AS plants, whereas its content in IS plants was significantly greater than that in AS plants (**Figure 2C**). There were no significant differences in leaf SOD activity, regardless of the treatment (**Figure 2D**). Pro content in the IS and AS plants was markedly higher than that in the control plants (**Figure 2E**).

The above parameters in roots differed from those in leaves. The H_2_O_2_ content in the roots of IS plants was significantly lower than that in both the control and AS plants (**Figure 2F**). CAT activity was significantly reduced in both IS and AS plants (**Figure 2G**). The MDA content was comparable between the control and IS plants, but lower in AS plants (**Figure 2H**). SOD showed the same trend as CAT (**Figure 2I**). The Pro content in IS and AS plants was significantly higher than that in control plants.

### 3.3 Proteomic changes in leaves and roots of *S. portulacastrum*

**Figure 3:**
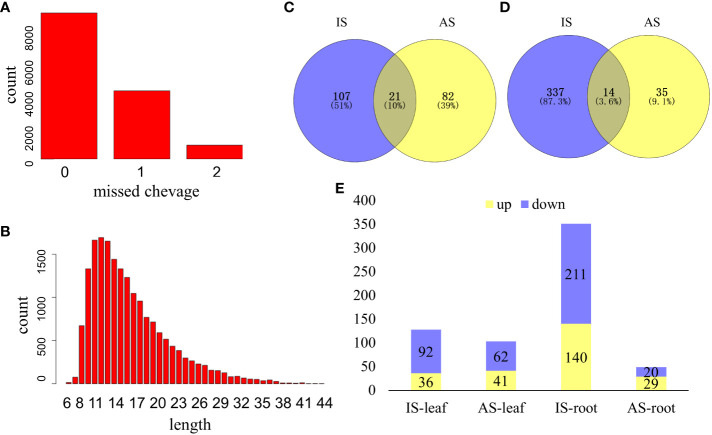
An overview of proteome profiles in the leaves and roots of *S. portulacastrum* plants after exposure to 0.5 M NaCl for 12 h as immediate stress (IS) and 14 days as adaptive stress (AS). **(A)** Protein missed cleavage; **(B)** proteins protein length distribution of proteome profile; **(C)** differentially expressed proteins (DEPs) in the leaves of IS and AS plants; **(D)** DEPs in the roots of IS and AS plants; and **(E)** upregulated and downregulated DEPs in different groups.

**Figure 4:**
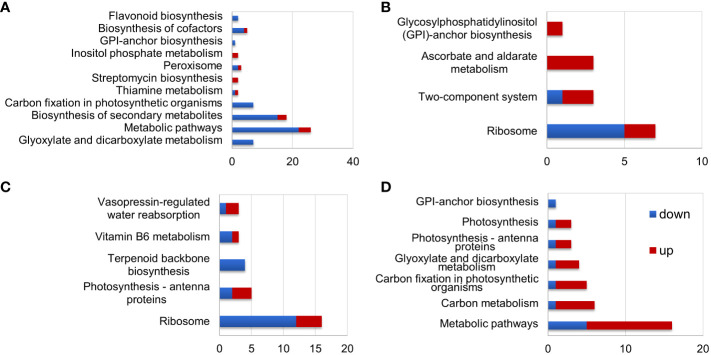
KEGG analysis of differentially expressed proteins (DEPs) in *S. portulacastrum* plants after exposure to 0.5 M NaCl for 12 h as immediate stress (IS) and 14 days as adaptive stress (AS). **(A)** DEPs in the leaves of IS plants, **(B)** DEPs in the leaves of AS plants, **(C)** DEPs in the roots of IS plants, and **(D)** DEPs in the roots of AS plants.

A total of 15,758 peptides belonging to 4,669 proteins were collected, and differentially expressed proteins (DEPs) were defined based on the foldchange ≥1.5 or foldchange ≤0.667, and *P* ≤0.05 from both the IS group and AS group (**Figure 3**). Missed protein cleavage is shown in **Figure 3A**, and the protein length distribution is presented in **Figure 3B**. There were 210 DEPs in leaves (**Figure 3C**) and 386 DEPs in roots (**Figure 3D**) of *S. portulacastrum*. In the leaves, 66.7% of DEPs were downregulated (**Figure 3E**). Root DEPs were almost two times higher than the DEPs in the leaves (**Figure 4**). We employed qRT-PCR to verify the trend of protein expression by detecting the relative expression of 10 protein-coding genes, and the expression trend of those genes was consistent with the trend of proteins (**Supplementary Figure 2**), which verified the reliability of the proteomic results.

#### 3.3.1 GO and KEGG enrichment analyses of DEPs

The leaf DEPs enriched in plants from IS and AS treatments were those involved in biological processes (BPs), cellular components (CCs), and molecular function (MF). The highly enriched DEPs in IS leaves were those involved in MF, mainly in oxidoreductase activity, cation binding, metal ion binding, and BP, followed by CC, such as those associated with plastid and chloroplast formation (**Supplementary Figure 3A**). DEPs highly enriched in AS leaves were those implicated in BP including organic substances and cellular biosynthesis processes, and then CC, but few in MF (**Supplementary Figure 3B**). DEPs enriched in the roots of IS plants were predominantly those engaged in BP including carbohydrate metabolic processes, oxidation–reduction processes, and photosynthesis. DEPs in the CC and MF were lower than those in the BP (**Supplementary Figure 3C**). Similarly, enriched DEPs in AS roots were mainly those involved in BP, with few enriched DEPs in both CC and MF (**Supplementary Figure 3D**).

KEGG pathway analysis showed that DEPs in the leaves of IS plants were primarily enriched in metabolic pathways, biosynthesis of secondary metabolites, and carbon fixation in photosynthetic organisms (**Figure 4A**). Enriched DEPs in the leaves of AS plants included those involved in ribosome, two-component system, and ascorbate and aldarate metabolism (**Figure 4B**). The DEPs enriched in the roots of IS plants included those implicated in ribosome and photosynthesis-antenna proteins (**Figure 4C**). Contrary to the fact that most DEPs in the roots of IS plants were downregulated, enriched DEPs in the roots of AS plants were mainly upregulated, which included metabolic pathways and carbon metabolism (**Figure 4D**). KEGG pathway analysis showed that more DEPs were enriched in the leaves of IS plants and roots of AS plants than in their corresponding counterparts. More importantly, DEPs in the roots were largely upregulated compared to those in the leaves. Short-term exposure to salt stress more significantly affected the aerial parts of plants, whereas relatively long-term stress required roots to adjust plant growth through metabolism-related proteins.

### 3.4 Overview of metabolomic results in *S. portulacastrum* roots

**Figure 5:**
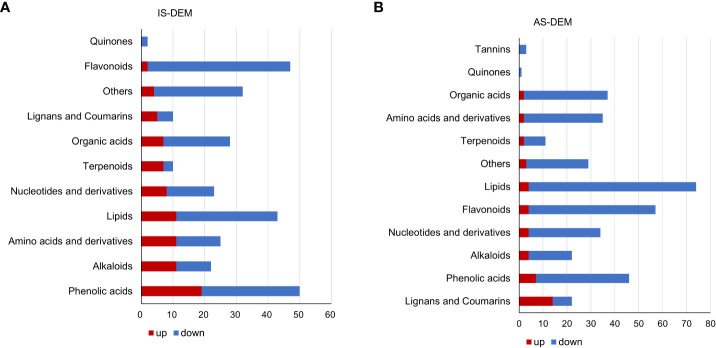
Differentially expressed metabolites (DEMs) of *S. portulacastrum* plants after exposure to 0.5 M NaCl for 12 h as immediate stress (IS) and 14 days as adaptive stress (AS). **(A)** Metabolite classes in the roots of IS plants and **(B)** metabolite classes in the roots of AS plants. The red bar represents upregulated DEMs, and blue represents downregulated DEMs.

Metabolomics of the control and salt-treated plant roots was analyzed. Metabolites with expression changes of more than 2-fold or less than 0.5-fold and a *P*-value ≤0.05, were defined as significant changes or differentially expressed metabolites (DEMs) (**Table 2**; **Supplementary Table S1**). There were 292 DEMs in the roots of IS plants. Among them, 70.9% were downregulated and 29.1% were upregulated. Most metabolites showed a downward trend, of which 45 DEMs belonged to flavonoids (21.7%), followed by lipids and phenolic acids (15% each). In the upregulated metabolites, the majority of them (19 DEMs) were related phenolic acid metabolism, following by alkaloids, lipids, and amino acids and derivatives, 11 DEMs each (**Figure 5A**).

**Table 2:** Differently expressed metabolites (DEMs) in roots of *portulacastrum* plants grown in one-fourth Hoagland solution after exposure to 0.4 M NaCl for 24 h as immediate stress (IS) and 14 days as adaptive stress (AP), respectively.

Group name	Differently expressed metabolites	Downregulated	Upregulated
IS	292	207	85
AS	371	325	46

A total of 371 DEMs were identified in the roots of AS plants. Downregulated DEMs accounted for 87.6%, and upregulated DEMs accounted for 12.4%. Among the downregulated DEMs, there were 70 DEMs related to lipid biosynthesis, representing the largest proportion at 21.5%, followed by 53 flavonoids accounting for 16.3%, and then 39 phenolic acids and 35 organic acids, 12% and 10.8%, respectively. In the upregulated metabolites, 14 lignans and coumarin DEMs, which accounted for the largest proportion at 30.4%. In addition, phenolic acids accounted for 15.2%, and nucleotides and their derivatives, flavonoids, alkaloids, and lipids accounted for 9% (**Figure 5B**).

The top 10 DEMs in the roots of IS plants with the largest upregulated fold change were saccharides and alcohols (Log2FC: 15.18), nucleotides and derivatives (Log2FC: 12.90), alkaloids (Log2FC: 12.45), phenolamine (Log2FC: 11.81), triterpene (Log2FC: 11.35), and phenolic acids (Log2FC: 11.11). While the top 10 DEMs with the largest downregulated fold change in roots of IS plants included anthraquinone (Log2FC: −14.57), organic acids (Log2FC: −12.72), phenolic acids (Log2FC: −11.89), free fatty acids (Log2FC: −6.44), vitamin (Log2FC: −4.51), and flavanols (Log2FC: −4.23) (**Supplementary Table S2**). The top 10 DEMs in the roots of AS plants with the largest upregulated fold change were phenolic acids (Log2FC: 13.25), flavanols (Log2FC: 2.63), nucleotides and derivatives (Log2FC: 2.53), flavones (Log2FC: 2.31), coumarins (Log2FC: 2.26), organic acids (Log2FC: 2.21), and saccharides and alcohols (Log2FC: 2.09). The top 10 DEMs with the largest downregulated fold change in the roots of AS plants included flavanols (Log2FC: −18.88), vitamins (Log2FC: −18.57), saccharides and alcohols (Log2FC: −16.48), organic acids (Log2FC: −15.18), nucleotides and derivatives (Log2FC: −14.36), amino acids and derivatives (Log2FC: −14.21), alkaloids (Log2FC: −12.92), and flavanones (Log2FC: −12.53) (**Supplementary Table S2**). The most enriched metabolites in the KEGG pathway with a *P*-value ≤0.05 in the roots of IS plants were metabolic pathways (metabolome frequency: 72.22%), biosynthesis of secondary metabolites (37.50%), and carbon metabolism (6.11%). In the roots of AS plants, the top three pathways (*P*-value ≤0.05) were the metabolic pathways (72.02%), purine metabolism (5.82%), and caffeine metabolism (1.11%) (**Supplementary Table S3**).

**Figure 6:**
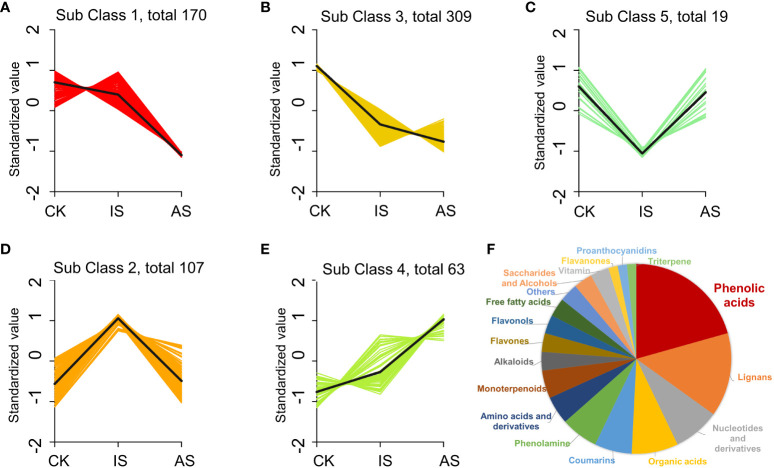
K-means cluster analysis of differentially expressed metabolites (DEMs) in change of relative contents. DEM changes in the roots of control, IS, and AS plants in relative contents and trends in the subclass 1 **(A)**, 2 **(B)**, 3 **(C)**, 4 **(D)**, and 5 **(E)**, as well as different proportions of DEMs in the class 4 **(F)**.

To further analyze the changes in metabolites, the relative contents of all DEMs were subjected to z-score normalization, followed by K-means cluster analysis (**Figure 6**; **Supplementary Table S4**). There were five subclasses of DEPs. Metabolites in subclasses 1 and 3 decreased in the roots of IS and AS plants (**Figures 6A, B**). Metabolites in subclass 5 showed a downregulation in IS roots and an upregulated trend in AS roots (**Figure 6C**), whereas metabolites in subclass 2 were opposite to those in subclass 5 (**Figure 6D**). DEMs in subclass 4 exhibited an upward trend (**Figure 6E**). Subclass 4 included mainly phenolic acids (20.63%), lignans (14.29%), nucleotides and derivatives (8%), and organic acids (8%) (**Figure 6F**). The phenolic acid metabolites were maleoyl-caffeoylquinic acid and chlorogenic acid (3-O-caffeoylquinic acid) in the roots of IS plants, and lignans and coumarin metabolite O-feruloyl 4-hydroxycoumarin in the roots of AS plants, which could be implicated in the regulation of *S. portulacastrum* tolerance to salt stress (**Supplementary Tables S2, S3**).

#### 3.4.1 Hormone signaling and phenolic acids synthesis and metabolism were crucial to plant salt tolerance

**Figure 7:**
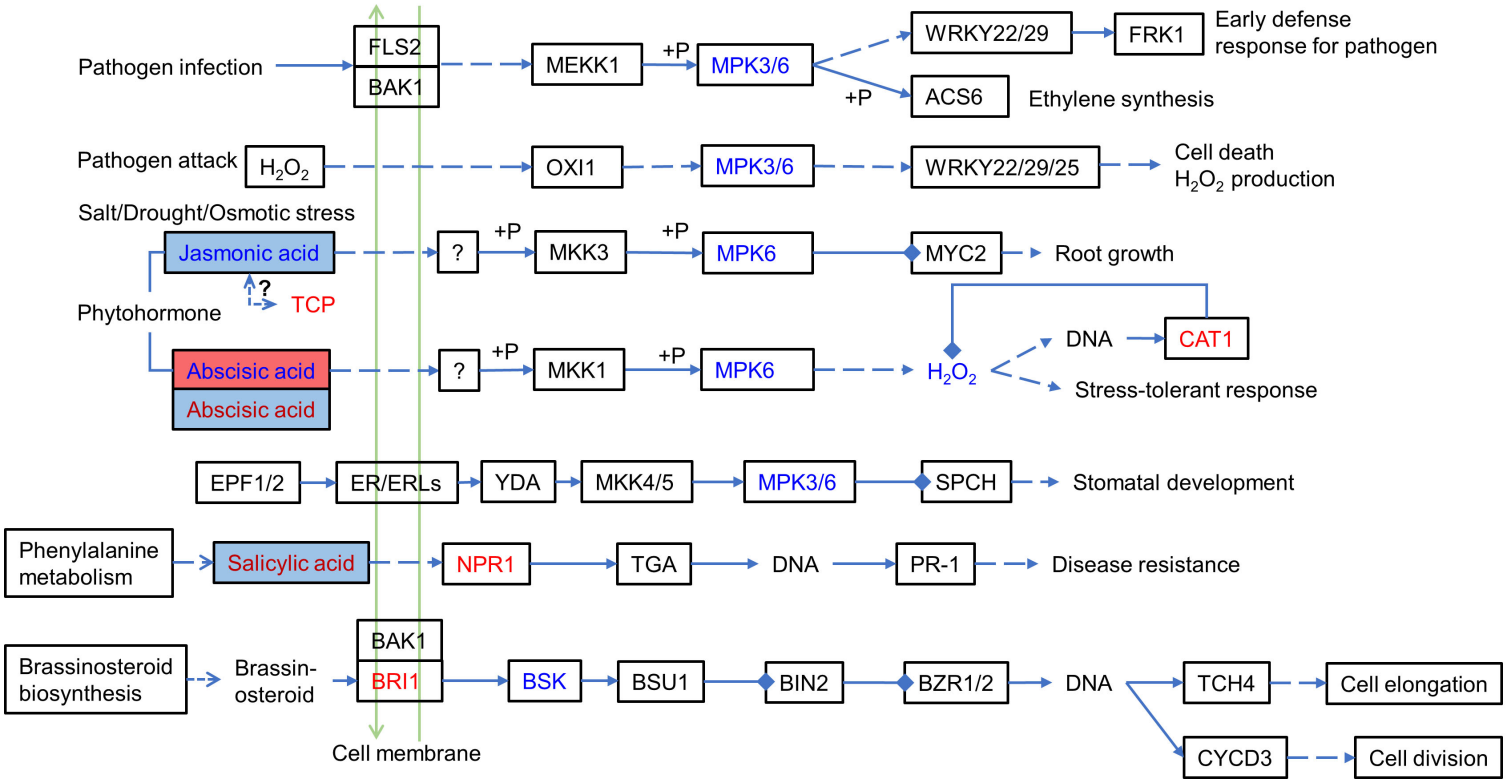
MAPK signaling pathway and phytohormone signal transduction involving differentially expressed proteins and metabolites. Red letters and blue letters represent up- and downregulated DEPs, respectively. Red and blue squares represent up- and downregulated DEMs, respectively. Inside the colored square, blue letter, red letter, and black letter represent DEMs occurring in IS, AS, both IS and AS plants, respectively. The solid line represents the relationship that has been established, the dashed line represents the putative regulatory relationship, the triangular arrow represents the positive regulatory role, and the four-square arrow represents the negative regulatory relationship. TCP, teosinte branched/cycloidea/PCF; NPR1, regulatory protein NPR1; BRI1, protein brassinosteroid insensitive 1; MPK3/6, mitogen-activated protein kinase 3/6; and CAT1, catalase 1.

Among the significantly correlated DEPs and DEMs, they had a significantly high correlation (r >0.8) with the hormones ABA (abscisic acid), JA (jasmonic acid), and precursors in hormone biosynthesis, including the auxin synthesis precursor tryptophan. For example, ABA (Lmtn004049) was upregulated (log2FC: 1.8613) in IS plants and downregulated (log2FC: −2.8446) in AS plants. JA (JA, pme1654) was downregulated (log2FC: −1.7825) in IS plants. The MAPK signaling pathway and phytohormone signal transduction were summarized by differentially expressed proteins and metabolites (**Figure 7**), which showed that these proteins and metabolites participated in the MAPK signaling pathway and phytohormone signal transduction. Phytohormones including JA, ABA, and salicylic acid (SA) regulate the salt adaptation process. The JA content was downregulated in IS plants, and ABA was first upregulated in IS plants and then downregulated in AS plants. SA is upregulated in AS plants. Phytohormones alerted downstream protein expression. CAT1, a negative regulator of H_2_O_2_, was also found to be upregulated. The downstream proteins of SA NPR1 and brassinosteroid downstream protein BRI1 were upregulated, whereas BSK was downregulated. Key factors of the MAPK signaling pathway, MPK3/6, were downregulated in the roots of IS plants.

**Figure 8:**
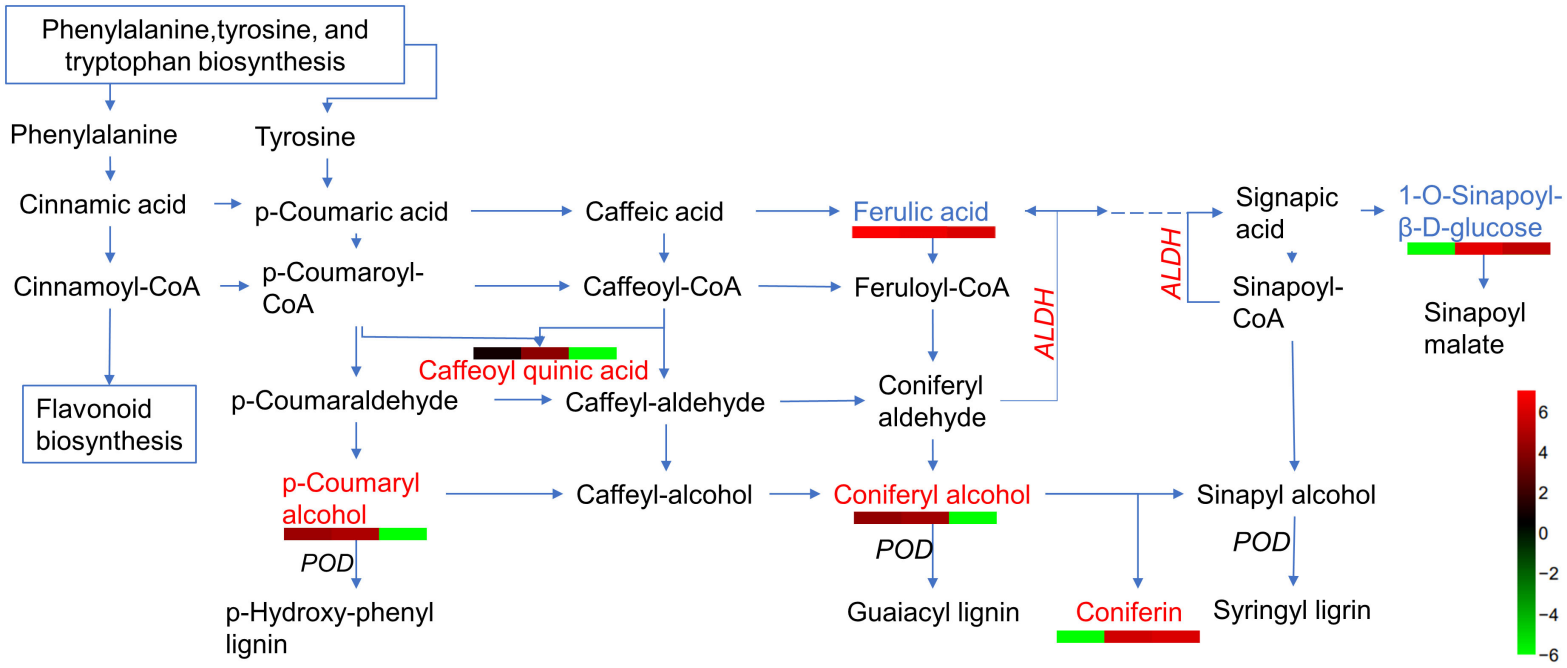
A simplified outline of metabolites and proteins differentially expressed in the phenolic acid biosynthetic pathway. Red letters indicate upregulated metabolites, blue letters are downregulated metabolites, italic letters represent differentially expressed proteins (DEPs), and dashed lines represent omitted partial metabolic processes. Heatmaps are drawn according to log10 of metabolite detection values. ALDH, aldehyde dehydrogenase, and POD, peroxidase.

**Figure 9:**
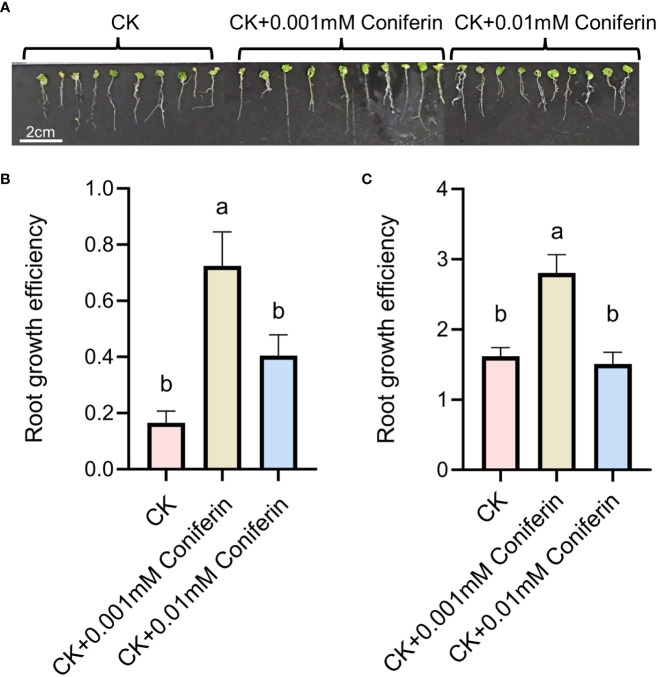
Root responses of *Arabidopsis thaliana* grown in a medium supplemented with 150 mM NaCl to two concentrations of coniferin. A depiction of plants grown on a medium with or without coniferin **(A)**. Root growth efficiency of plants cultivated on the medium for seven days **(B)** and 14 days **(C)**. The letters above bars represent significant differences analyzed by LSD at *P<*0.001 level.

Based on the analysis of metabolites combined with the analysis of proteins, differentially expressed proteins and metabolites from the same treatments were simultaneously mapped to the KEGG pathway to explore the relationship between proteins and metabolites. In the IS plants, there were 321 metabolites and 248 proteins that were significantly correlated. These proteins and metabolites were enriched 35,816 times in 203 KEGG pathways (**Supplementary Table 5**). In the AS plants, 395 metabolites were significantly correlated with 27 proteins; they were enriched 3,354 times in 23 KEGG pathways, including carbon metabolism, biosynthesis of amino acids, ABC transporters, carbon fixation in photosynthetic organisms, glyoxylate and dicarboxylate metabolism, and 2-Oxocarboxylic acid metabolism. Interestingly, most of the upregulated proteins and metabolites were enriched in the phenolic acid synthesis and metabolism pathways (**Figure 8**; **Supplementary Table S6**). Among these, coniferin appears to play a positive regulatory role in plant salt tolerance. To verify the effect of phenolic acid on salt tolerance, coniferin was selected to test if *A. thaliana* seedlings became more tolerant of salt stress when grown in a medium containing 150 mM NaCl (**Figure 9A**). Results showed that the addition of 0.001 mM coniferin to the medium significantly increased root growth rates of *Arabidopsis* after seven (**Figure 9B**) and 14 days (**Figure 9C**) of transplanting.

## 4 Discussion

As a halophyte, *S. portulacastrum* has been increasingly studied, including its morphological and physiological responses to salt stress (Wang et al., 2012; Yi et al., 2014; Ding et al., 2022; He et al., 2022), leaf proteomic and metabolomic changes after exposure to salt, and molecular analysis of its tolerance mechanisms (Wang D. et al., 2022). Additionally, *S. portulacastrum* has a remarkable capacity to abate environmental pollutants (Zhang et al., 2022). When *S. portulacastrum* is used to remediate seawater contaminants, the roots come directly in contact with various pollutants. Roots have to first respond to salt stress and then communicate with shoots to activate defense mechanisms to mitigate adverse effects. Thus, this study placed a major emphasis on roots and analyzed proteomic changes in roots and leaves as well as metabolomic changes in the roots of *S. portulacastrum* after 24 h and two weeks of exposure to salt stress, respectively. We found that there were more DEPs in the roots than in the shoots, and most of them were involved in metabolic pathways. Metabolomic analysis of roots showed that a large number of DEMs occurred in the roots, of which phenolic acids were highly regulated and could play an important role in salt stress tolerance of *S. portulacastrum*.

### 4.1 Morphological and physiological responses to salt stress

Previous studies have shown that the effects of salt stress on root growth depend on the severity of stress levels and the plant species (Duan et al., 2013; Geng et al., 2013). In the present study, the root length of AS plants increased significantly compared with the control, suggesting that *S. portulacastrum* plants were able to adapt to 0.4 M NaCl (**Figure 1**). Na^+^ affects photosynthesis by disrupting proton dynamics, chloroplast function, and CO_2_-fixing enzymes (van Zelm et al., 2020). Chlorophyll content represents the photosynthetic capacity (Croft et al., 2020). Our study showed that, compared with the AS plants, the chlorophyll content was significantly reduced only in the IS plants, and the GO category of “photosynthesis” was only enriched in the IS plants, indicating that the photosynthetic ability of AS plants was restored in two weeks. These results are consistent with those of a previous study showing that *S. portulacastrum* adapted to salt stress in a short period of time (Wang D. et al., 2022).

Plants show multiphase responses to salt stress, from salt sensing to early signaling and ion transport (van Zelm et al., 2020). Salt stress initially triggers Ca^2+^ and ROS signaling, followed by phospholipid and protein kinase signaling, which then activates proton pumps to initiate cellular ion transport and maintain sodium/potassium homeostasis (Fan et al., 2017). The overaccumulation of H_2_O_2_ can damage cells under abiotic stress (Habibi, 2014), and plants quickly activate antioxidant defense enzymes to reduce oxidative effects. MDA is a lipid peroxidation marker that indicates the degree of plasma membrane damage and ability to tolerate stress. SOD converts superoxide radicals (O_2_^−^) into H_2_O_2_ (Kumari et al., 2015). After salt treatment, SOD levels in the roots and leaves of *S. portulacastrum* changed slightly, indicating that the salt concentration in this study did not damage the ROS homeostasis system (Wang D. et al., 2022). Compared with glycophytes, MDA and H_2_O_2_ contents in *S. portulacastrum* were higher than those in water dropwort (*Oenanthe javanica*) and rice after salt treatment (Sun et al., 2010; Kumar et al., 2021), suggesting that halophytes have a greater tolerance to ROS than glycophytes. MDA content in roots was higher than that in leaves (**Figure 2**), and the changes in antioxidant enzymes and MDA content in leaves differed from those in roots, indicating that differential responses to salt stress occurred in *S. portulacastrum* roots and leaves (Fan et al., 2017; Yang and Guo, 2018). CAT is an important enzyme for H_2_O_2_ removal (Yang and Guo, 2018), and its activity differs between roots and leaves (Sun et al., 2010; Kumar et al., 2021) in different cultivated species of the same genus (Mekawy et al., 2020). In this study, CAT in leaves showed a trend of increase in IS but a significant decrease in AS, while it decreased in both IS and AS by the same magnitude (**Figure 2**), suggesting that roots of *S. portulacastrum* were more tolerant to H_2_O_2_ than leaves.

Accumulation of osmolytes is another strategy used by plants to tolerate salt stress. There are two types of osmolytes: inorganic ions and organic solutes. Inorganic ions such as K^+^ and organic solutes such as glycine betaine, proline, soluble sugars, polyols, and polyamines play important roles in plant salt tolerance (Zivcak et al., 2016). Under drought stress, proline is synthesized in the leaves and then transported to the roots in watermelon (Wang Z. et al., 2022). Under salt stress, we found that proline contents increased in the leaves and roots of IS and AS plants, but root proline in IS plants was higher than that in AS plants, suggesting the osmotic regulator proline in plant leaves and roots was slightly different, which is similar to chickpea (*Cicer arietinum* L.) (Hirich et al., 2014). Alanine betaine in *Populus euphratica* showed high accumulation in the leaves under salt and heat stress (Brosché et al., 2005). In the present study, D-proline betaine (Rfmb318) was upregulated in the roots of IS (log2FC: 1.9397) and AS (log2FC: 1.0829) plants, while alanine betaine (Amxp00801) and betaine (mws0191) showed a downregulated trend in the roots of AS plants (**Supplementary Table 5**). The osmolytes showed a downward trend within two weeks of salt treatment, which proved that *S. portulacastrum*, especially its root system, can fully adapt to the 0.4 M salt stress in two weeks. Previous studies on the root salt tolerance of *S. portulacastrum* found that differentially expressed genes related to signal transduction and transcription (genes encoding 14-3-3 like protein, ARF GTPase family protein, and H^+^-ATPase), stress/defense responses (transcription factor ASR5, ortholog of OsASR5), transport, and metabolism (genes encoding various ATPases, including P-ATPase, V-ATPase, inorganic PPase1, and ATPase subunit, F1) were upregulated under salt treatment (Fan et al., 2017). In the present study, salt stress induced expression of DEPs related to signal transduction and metabolism, including pyrroline-5-carboxylate reductase, voltage-gated shaker-like K^+^ channel proteins, and ATPase. Pyrroline-5-carboxylate reductase (P5CR) is a crucial enzyme in the proline synthesis pathway that regulates proline accumulation in plants (Vinocur and Altman, 2005), and its expression of antisense P5CR led to abiotic stress sensitivity in soybeans (De Ronde et al., 2004). In the present study, PC5R was upregulated in the leaves of AS plants, indicating halophyte tolerance to salinity. ATG3-dependent autophagy acts as an executor of mitochondrial remodeling to maintain homeostasis in the internal environment by removing ROS (Liu et al., 2016). In the present study, SpATG (A0A0K9RAJ3) was also differentially regulated, which indicates that *S. portulacastrum* may initiate the adaptation to salt stress after 24 h (**Supplementary Table 4**).

Ion exchange between extracellular and intracellular compartments is vital for plant adaptation to salt stress (Ding et al., 2022). The leaves of *S. portulacastrum* can compartmentalize salt ions, such as by transporting Na^+^ into the vacuole through a proton pump (Wang et al., 2012; Yi et al., 2014; Wang D. et al., 2022), which is an energy-consuming process that relies on vacuolar H^+^-ATPase (Apse et al., 1999). In *S. portulacastrum* leaves, vacuolar H^+^-ATPase V0 sector subunits were upregulated in both IS and AS plants, and AAA^+^-type ATPase was upregulated in IS, but downregulated in AS plants. In *S. portulacastrum* roots, F0F1-type ATP synthase alpha subunit and AAA^+^-type ATPase were upregulated in IS plants, whereas vacuolar H^+^-ATPase V1 sector, subunit C was downregulated in IS plants. Plasma membrane H^+^-ATPase was downregulated in AS plants. The results indicated that plants need more ATP for ion exchange and adaptation to salt stress during the initial stage of salt stress, indicating that ATPase might be more active in IS than in AS plants. NaCl treatment disrupts the Na^+^/K^+^ balance of plant cells and hinders the plant absorption of other ions (such as K^+^) (Nieves-Cordones et al., 2016). Voltage-gated shaker-like K^+^ channel subunit beta (KCNAB) was upregulated in AS plants and did not significantly change in IS plants, which showed that NaCl treatment affected K^+^ exchange. The expression of ion-exchange-related enzymes reflects the ability of plants to adapt to salt stress (Yi et al., 2014; Wang D. et al., 2022). AAA^+^-type ATPase in IS plants was upregulated at 7.5-fold in leaves and 2.31-fold in roots, while downregulated in AS plants. Leaf vacuolar H^+^-ATPase was upregulated in IS plants at 1.99-fold in leaves and 1.66-fold in AS plant leaves. Root F0F1-type ATP synthase was upregulated at 2.42-fold in IS and 1.78-fold in AS plants (**Supplementary Table 4**). Our study showed that *S. portulacastrum* could adapt to salt treatment within two weeks, which reflects the ability of *S. portulacastrum* to tolerate salt stress.

### 4.2 Salt induced signal transduction and regulation proteins and metabolites

Salt adaptation is a complex process, during which regulators play vital roles, including various signal transduction hormones and regulation-related proteins. Previous studies have shown that the salt-induced growth arrest stage is regulated by hormone levels, mainly ABA levels and ABA signaling transcripts (Geng et al., 2013). In the next recovery stage, ABA concentration decreased, whereas jasmonic acid (JA), brassinosteroid (BR), and gibberellic acid (GA) levels increased, and downstream transcription processes were induced. Endogenous JA plays a role in the rapid response to salt stress in glycophytes, such as *A. thaliana* (Ellouzi et al., 2013) and tomato (*L. esculentum*) (Pedranzani et al., 2003), whereas the JA content of the salt-tolerant plant *Medicago truncatula* showed no significant change (De Domenico et al., 2019; Ruan et al., 2019). In *S. portulacastrum* roots, JA content was significantly decreased in IS plants, which was negatively associated with salt treatment and was different from that of glycophytes (De Domenico et al., 2019; Ruan et al., 2019). *TCPs* (teosinte branched/cycloidea/PCF) are plant transcription factors involved in regulating plant form and architecture, cell cycle, and hormone signaling (Cubas et al., 1999; Martín-Trillo and Cubas, 2010). They can regulate enzymes in JA biosynthesis and influence JA levels in plants. Silencing cotton GbTCP led to lower JA levels (Schommer et al., 2008; Hao et al., 2012). In *S. portulacastrum*, the chaperonin complex component, TCP-1 alpha subunit (CCT1) (A0A0K9RDE9), was upregulated (Log2FC: 1.257) in the roots of IS plants, and JA content in roots was downregulated (**Supplementary Table 4**). The expression of SpTCP in *S. portulacastrum* was opposite to that in cotton. SpTCP may regulate JA content to help *S. portulacastrum* adapt to salt stress. Crosstalk exists between JA and ABA signal transduction in chlorophyll degradation and anthocyanin biosynthesis in Arabidopsis (Yip Delormel and Boudsocq, 2019; Yu et al., 2021). In the present study, ABA (Lmtn004049) was upregulated (log2FC: 1.8613) in IS plants and downregulated (log2FC: −2.8446) in AS plants (**Supplementary Table 5**). In Arabidopsis, the ABA-responsive element (ABRE)-binding factor ABF4 is phosphorylated by AtCPK32 (Choi et al., 2005) and AtCPK4 activates ABF2 upon ABA perception (Lu et al., 2013). Calreticulin (TaCRT1) plays an essential role in restraining salt stress in wheat (*Triticum aestivum*) (Xiang et al., 2015). A previous study reported that CRT, along with calcium-dependent protein kinase 13 (CDPK13) and calreticulin interacting protein 1 (CRTintP1), might be critical signaling factors in the response to abiotic stress in rice (Komatsu et al., 2007). In addition, CDPK genes can promote flavonoid biosynthesis under salt stress in the glycophyte *Glycyrrhiza uralensis* (Tong et al., 2019). We found that SpCRT (O81919) was upregulated (Log2FC: 1.3408) in the leaves of AS plants and SpCPK (A0A7C9D457) was downregulated (Log2FC: −1.2763) in the roots of IS plants (**Supplementary Table 4**). In this network, calcium-dependent protein kinases and their relative (CDPK/CPK) were involved in salt tolerance. When salt stimulates the lateral root, the Ca^2+^ signal can channel from the cortical and endodermal cell layers to the main root and aerial parts of the plant (Choi et al., 2014). Salt treatment transmits Ca^2+^ signals to CPK proteins, which are gradually downregulated as salt stress progresses, and calreticulin is gradually upregulated. A crosstalk between calcium-dependent protein kinase and MAPK (mitogen-activated protein kinase) signaling controls plants stress responses (Ludwig et al., 2005). MKK-MPK cascades, together with phytohormones, are involved in ROS homeostasis (Pitzschke et al., 2009) and play a vital role in signal transduction in plants under salt stress (Mantri et al., 2012).

**Figure 10:**
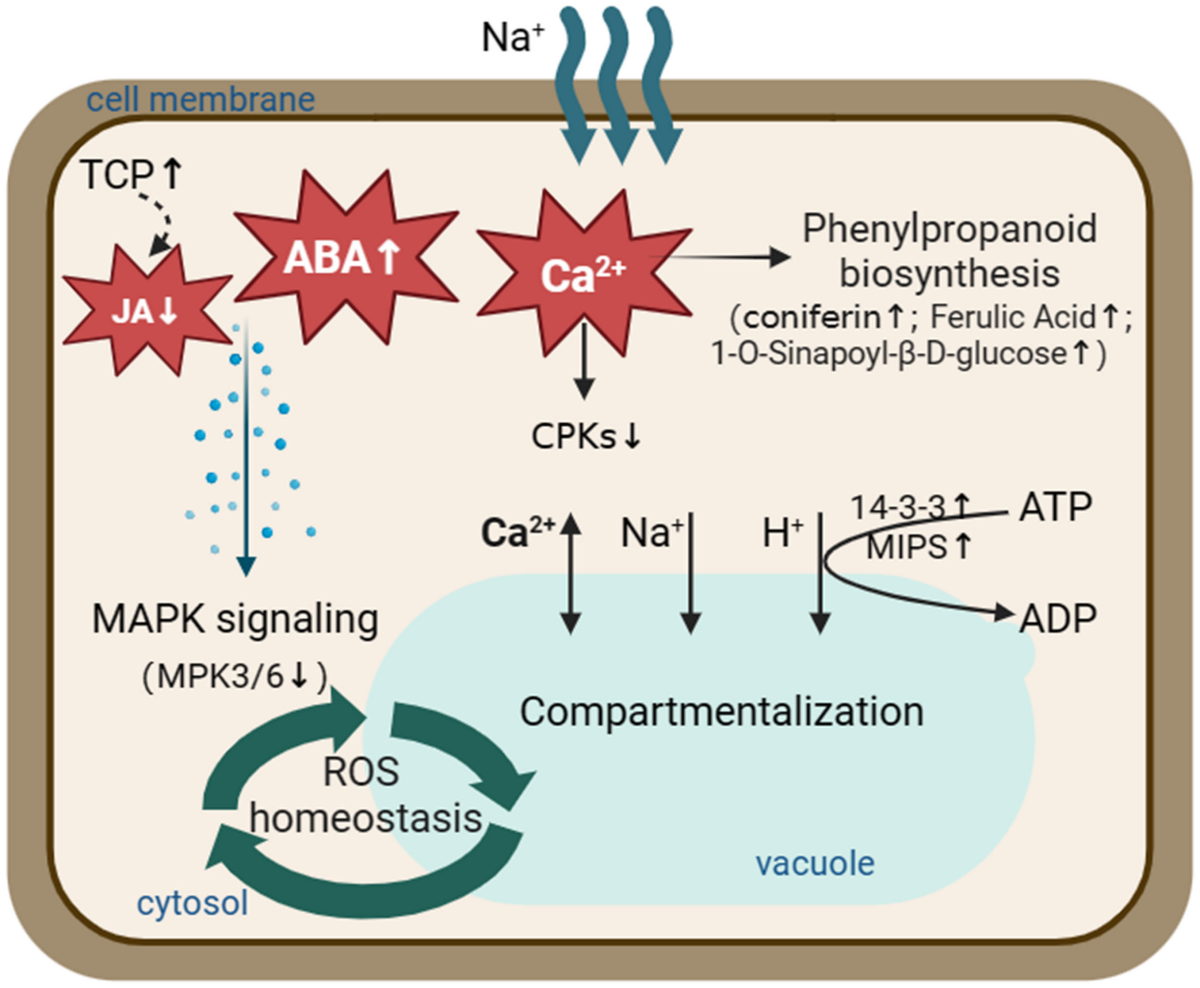
A schematic model illustrating proteins and metabolites involved in the salt tolerance in *S. portulacastrum* roots. Salt stress activates the phenylpropanoid biosynthesis pathway through Ca^2+^ signal transduction and increases the content of metabolites, such as coniferin. At the same time, it inhibits the MAPK signaling pathway through ABA and JA signal transduction and promotes the compartmentation of Na^+^ in the vacuole. Thus, plants could maintain ROS homeostasis and adapt to salt stress. The up arrows represent increased expression of the relevant proteins or metabolites. The down arrows represent relevant proteins or metabolites that are reduced expression.

Additionally, 14-3-3 proteins can bind to and activate CPK, and 14-3-3 genes are involved in the ABA signaling pathway in Arabidopsis (Xu et al., 2018). 14-3-3 proteins, also known as general regulatory factor (GRF) proteins, are ubiquitously found in eukaryotic organisms and play important roles in the response to stress stimuli (Ferl, 1996; Lyu et al., 2021). 14-3-3 proteins and MIPS (myo-inositol-1-phosphate synthase) promote various metabolites to confer salt tolerance. Overexpression of the MIPS gene can protect plants from abiotic stress by increasing various metabolites of basal metabolism, including glycolysis, the pentose phosphate pathway, the tricarboxylic acid cycle, and inositol metabolism, and confers salt tolerance in rice (Kusuda et al., 2015). Three days of saline exposure induced the transcription and enzyme activity of AdMIPS in the leaves, phloem, and roots of kiwifruit, whereas a longer period (more than15 days) resulted in the opposite effect, indicating that AdMIPS is salt-sensitive (Cui et al., 2013). Co-expression of D-MIPS and 14-3-3-like protein GF14 is responsible for the crosstalk between *Zea mays* and *Rhizophagus intraradices* under drought stress (Li et al., 2016). In this study, MIPS proteins (A0A7C9CZ99 and Q944C3) were detected in both the leaves and roots of IS plants with upregulating trends, and 14-3-3 (A0A0J8BBQ5) was upregulated (log2FC: 2.2483) in the roots of IS plants (**Supplementary Table 4**). In *S. portulacastrum*, MIPS, together with 14-3-3, was upregulated in roots after salt treatment, indicating their positive roles in salt adaptation. A hypothetical network was proposed involving JA and ABA hormones and proteins (**Figure 10**). The crosstalk between JA and ABA may be critical for halophyte salinity adaptation, which requires further investigation.

### 4.3 Phenolic acid metabolites and derivatives contribute to salt tolerance of *S. portulacastrum*

Phenolic acids are among the plants’ first lines of defense against abiotic and biotic stresses (Beckman, 2000). Phenolic acid exerts antioxidant activity, acts as a chain-breaking antioxidant, and plays an important role in altering cell signaling pathways (Chandrasekara, 2019). Phenolic acid metabolites and their derivatives, including FA, 1-O-Sinapoyl-β-D-glucose, and coniferin, were upregulated in salt-stressed *S. portulacastrum*.

Polyphenolic compounds can increase antioxidant activities under short-term salt stress by upregulating ferulic acid (FA), salicylic acid (SiA), kaempferol (KAE), and quercetin (QUE) content in Chinese cabbage (Linic et al., 2021). In this study, FA was highly expressed under salt treatment, which is consistent with the resulst of a previous study. Sinapoyl malate is an important metabolite related to UV-B absorption in plants (Kusano et al., 2011). The present study found that 1-O-sinapoyl-β-D-glucose, the precursor synthesis of sinapoyl malate, was upregulated by salt treatment.

Recently, coniferin was reported to play an important role in salt tolerance in plants (Chun et al., 2019). Coniferin is a coniferyl alcohol-β-D-glycoside, that is a dominant metabolite in conifers and has been reported in other plants (Hosel et al., 1982). Salt-tolerant suspension-cultured *Alluaudiopsis marnieriana* cells were able to accumulate coniferin under salt stress (Yoshioka et al., 2021). Coniferin was markedly increased in salt-adapted A120 cells of Arabidopsis (Chun et al., 2019). Coniferin in *S. portulacastrum* was highly upregulated. Additionally, Arabidopsis grown in medium supplemented with 0.001 mM coniferin became more tolerant to salt stress than control plants (**Figure 9**), indicating that coniferin may be a primary metabolite contribute salt tolerance in *S. portulacastrum*.

## 5 Conclusion

In the present study, the morphological and physiological responses of *S. portulacastrum* to different NaCl concentrations were investigated. Plants were able to grow in a hydroponic solution supplemented with 0.4 M NaCl without significant reduction of leaf and root numbers, canopy height, or relative growth rates. Subsequently, plants grown at this Na concentration for 24 h and two weeks were used for studying immediate and adaptive salt stress at proteomic and metabonomic levels. A total of 47 DEPs were identified in leaves, and 248 DEPs occurred in roots. KEGG analysis showed that DEPs, especially root-DEPs, were highly enriched in the metabolic pathways. Metabonomic analysis enriched 292 DEMs in the roots of IS plants and 371 DEMs in the roots of AS plants. Signal transduction, phenolic acid synthesis, and metabolism are crucial for *S. portulacastrum* to tolerate salt stress. Through integrative analysis of proteomics and metabolomics, a working model was proposed to illustrate the integrative responses of *S. portulacastrum* to salt stress. Specifically, salt stress activated Ca^2+^, ABA, and JA signaling networks, which promoted the phenylpropanoid biosynthesis pathway through Ca^2+^ signal transduction and increased the content of metabolites such as coniferin, while salt stress inhibited the MAPK signaling pathway through ABA and JA signal transduction and promoted the sequestration of Na^+^ to the vacuole. *S. portulacastrum* plants were able to maintain ROS homeostasis and adapt to salt stress (**Figure 10**).

## Data availability statement

The mass spectrometry proteomics data was deposited to the ProteomeXchange Consortium (http://proteomecentral.proteomexchange.org) via the iProX partner repository (Ma et al., 2019; Chen et al., 2022a) with the dataset identifier: PXD044063.

## Author contributions

DC: Conceptualization, Data curation, Methodology, Writing – original draft, Writing – review & editing. WZ: Conceptualization, Investigation, Writing – review & editing. NY: Data curation, Software, Visualization, Writing – original draft. ZL: Data curation, Investigation, Methodology, Writing – review & editing. CZ: Data curation, Methodology, Software, Validation, Writing – review & editing. DW: Formal analysis, Methodology, Supervision, Writing – review & editing. GY: Data curation, Supervision, Validation, Visualization, Writing – review & editing. JC: Conceptualization, Visualization, Writing – review & editing, Supervision, Validation. XW: Funding acquisition, Resources, Writing – review & editing, Supervision.
