# Supplementary material for: Proteomic and metabolomic analyses uncover integrative mechanisms in Sesuvium portulacastrum tolerance to salt stress
Source: Front Plant Sci. 2023 Nov 28;14:1277762. doi: 10.3389/fpls.2023.1277762 (PMC10714944; doi:10.3389/fpls.2023.1277762)
Supplement: Supplementary file 1 [file DataSheet_1.docx]

Supplementary Materials

# Supplementary Figures

**
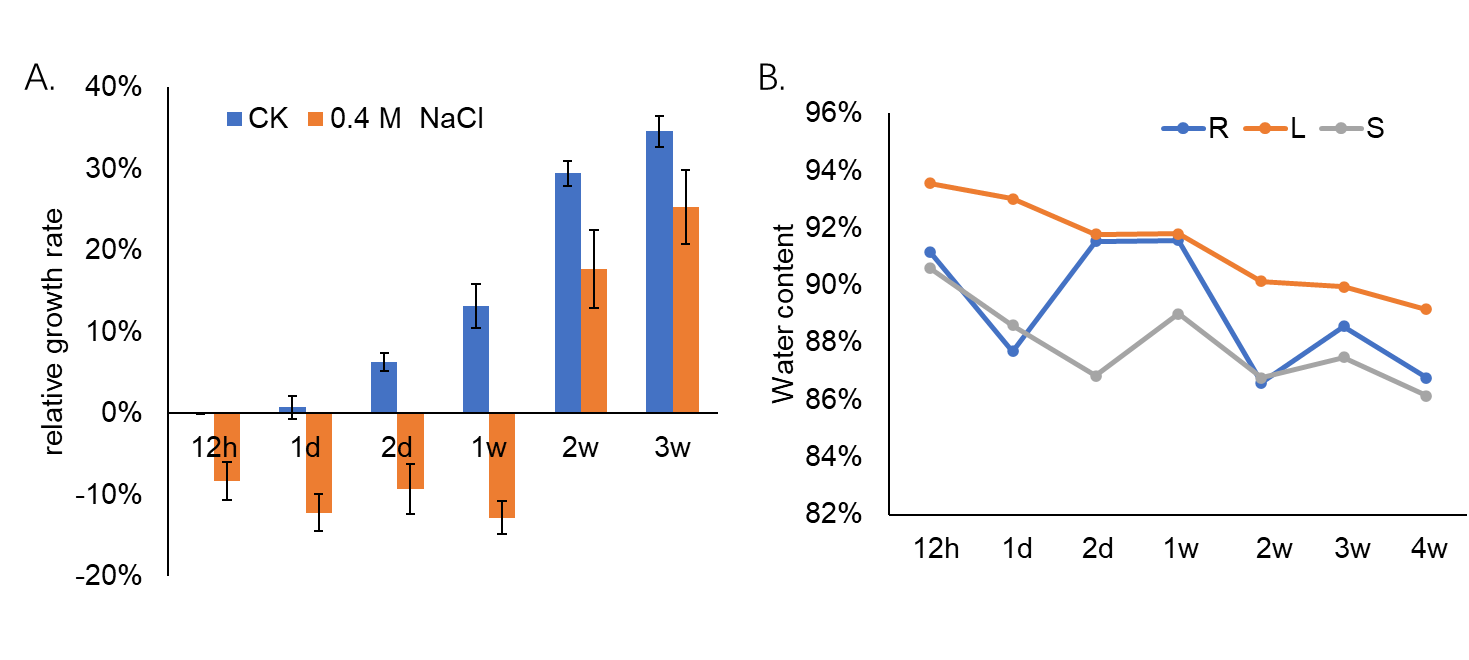
**

**Supplementary Figure S1.** The relative growth rate and water content of *Sesuvium portulacastrum* plants grown in a ¼ Hoagland solution supplemented with 0.4 M NaCl over a period of time. (A) Relative growth rate of control (CK) and treated plants (0.4 M NaCl) in 12 h, one day to three weeks. (B) Water content of leaves, stems, and roots of salt stressed (0.4 M NaCl) plants grown in the solution in 12 h, one day to four weeks. Bars in (A) represent standard error.


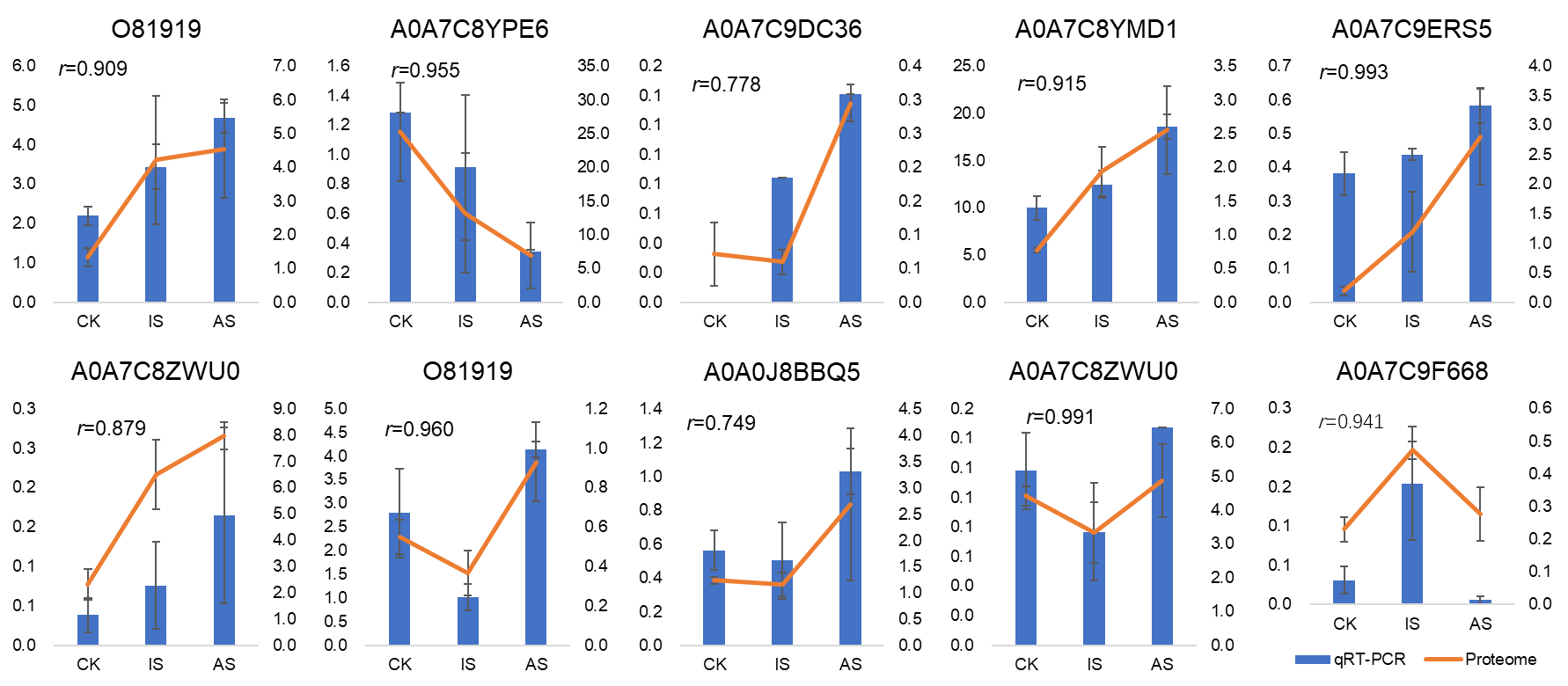


**Supplementary Figure S2.** Relative expression levels of differentially expressed proteins identified through proteomic analysis and qRT-PRC analysis of relative expression levels of genes encoding these DEPs. Blue bars and left Y-axis indicate the relative expression of proteins, and orange line and right Y-axis represent gene expression. *Sesuvium portulacastrum* plants grown in a ¼ Hoagland solution without Na as control (CK) and those grown in 0.4 M NaCl for 12 h as the immediate salt stress response (IS) and for two weeks as the adaptive salt stress response (AS). Titles are the protein ID. *r* is the correlation coefficient.


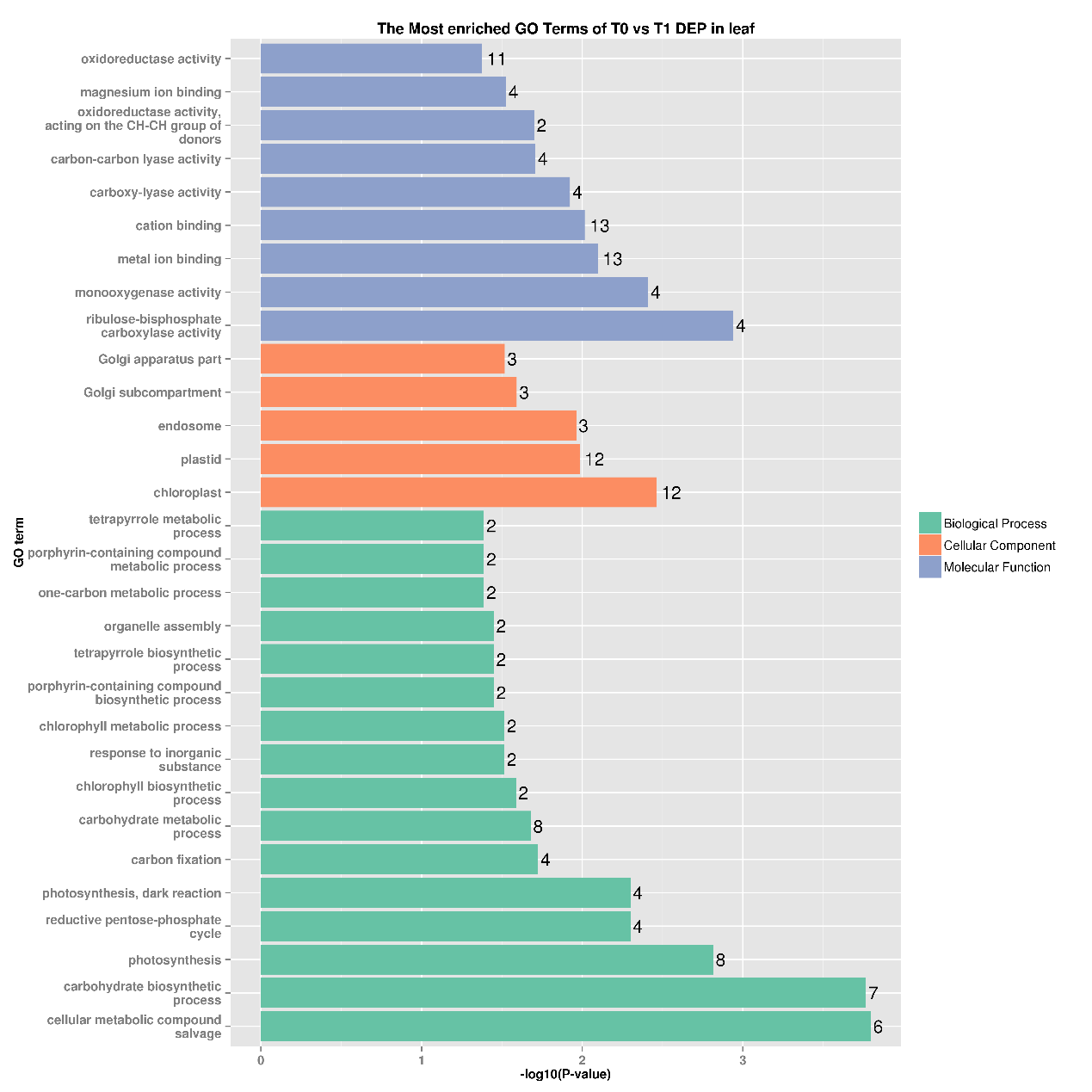


A


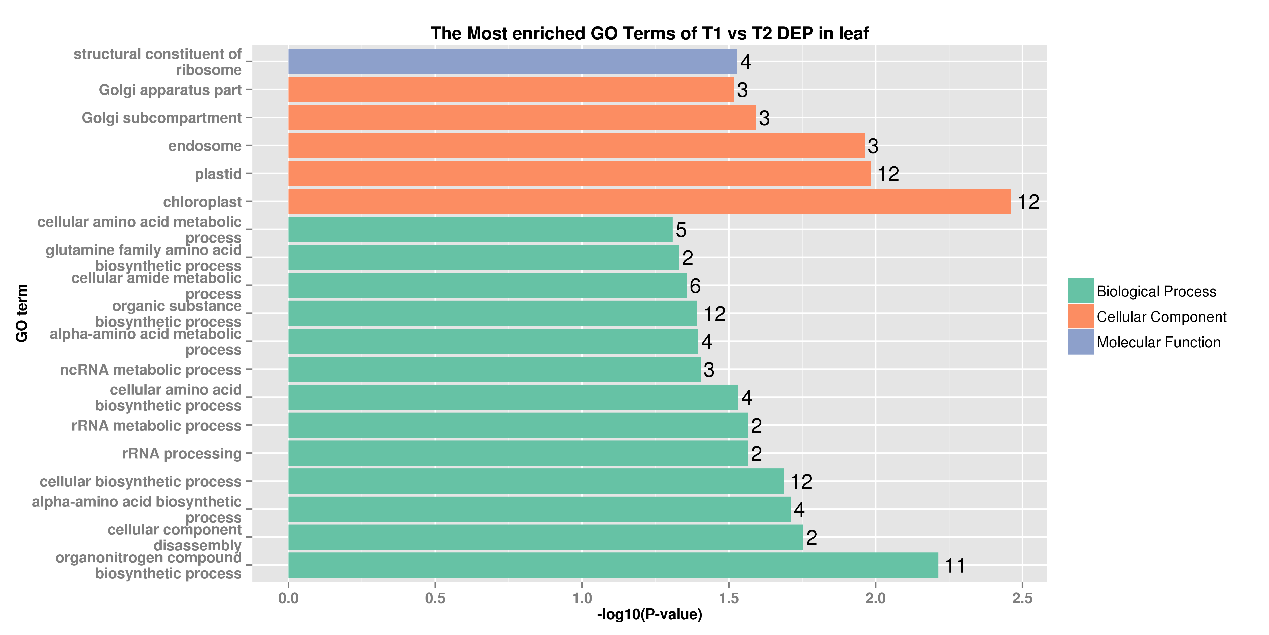


B


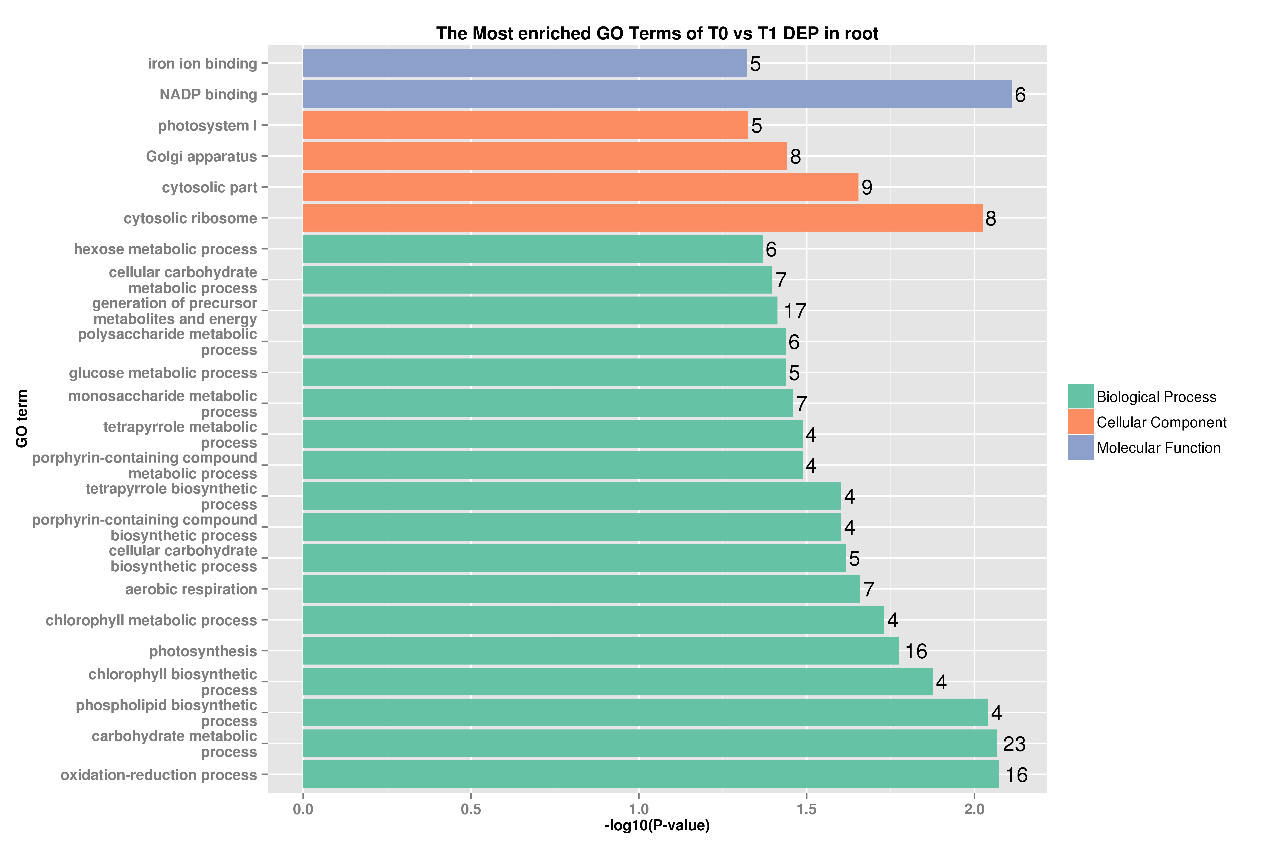


C


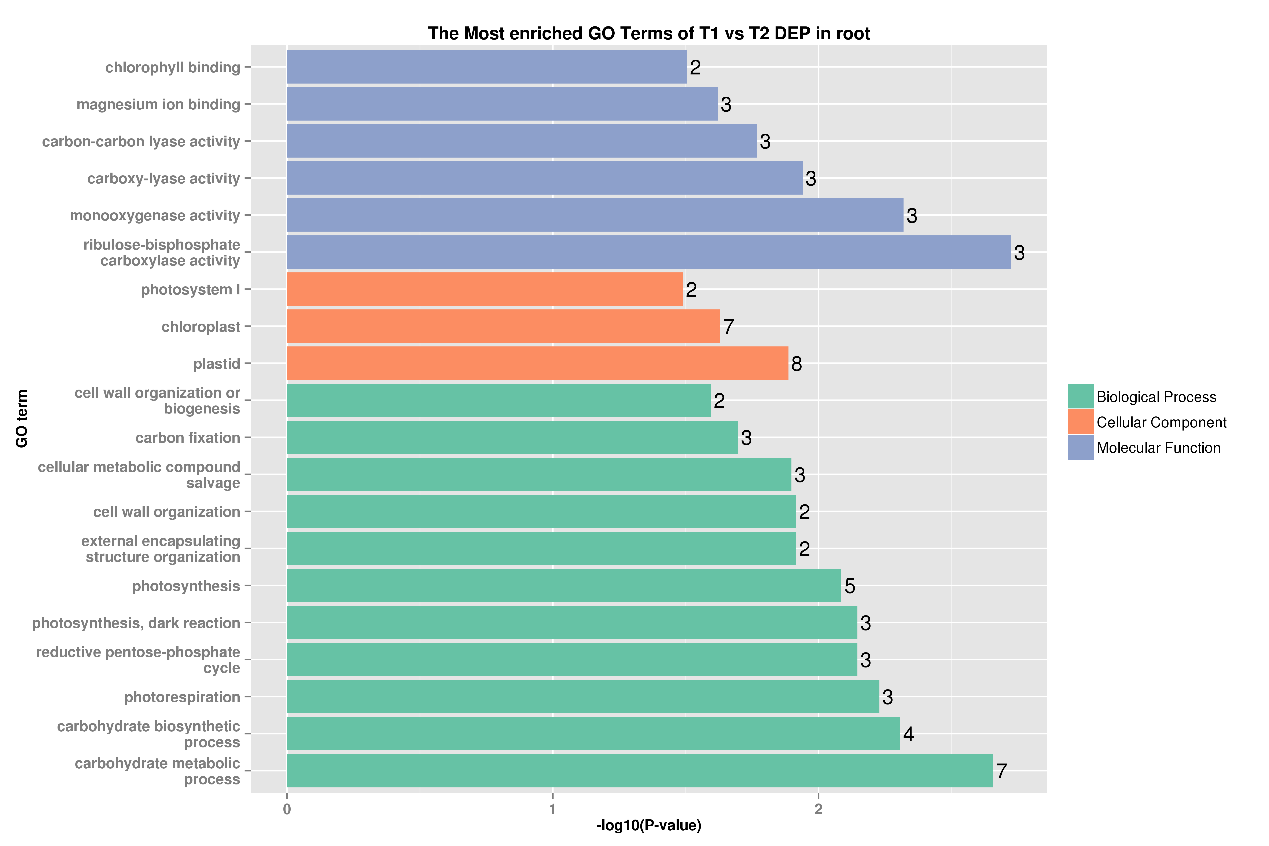


D

**Supplementary Figure S3.** The most enriched GO terms of differentially expressed proteins (DEPs) in leaves and roots of *Sesuvium portulacastrum* plants grown in a ¼ Hoagland solution supplemented with 0.4 M NaCl for 12 h as immediate salt stress response (IS) and two weeks as adaptive salt stress response (AS). The most enriched GO terms of DEPs in leaves of IS (A) and AS plants (B), and the most enriched GO terms of DEPs in roots of IS (C) and AS (D) plants.
